# Supplementary material for: Genetic Effects of LPIN1 Polymorphisms on Milk Production Traits in Dairy Cattle
Source: Genes (Basel). 2019 Apr 2;10(4):265. doi: 10.3390/genes10040265 (PMC6523124; doi:10.3390/genes10040265)
Supplement: Supplementary file 1 [file genes-10-00265-s001.pdf]

Table S1-1. Descriptive statistics of the phenotypic values for milk production traits in the first lactation.

Traits	Mean	S.D.	Min.	Max.	C.V.
Milk yield (kg)	10379.81	1476.18	6057.96	14505.68	14.22
Fat yield (kg)	350.55	60.7	184.8	537.97	17.31
Fat percentage (%)	3.387	0.423	2.059	4.742	12.49
Protein yield (kg)	313.78	48.2	157.73	457.53	15.36
Protein percentage (%)	3.024	0.198	2.257	3.508	6.548

Table S1-2. Descriptive statistics of the phenotypic values for milk production traits in the second lactation.

Traits	Mean	S.D.	Min.	Max.	C.V.
Milk yield (kg)	10859.85	1876.61	4756.94	16512.08	17.28
Fat yield (kg)	390.12	83.85	145.05	658.25	21.49
Fat percentage (%)	3.599	0.504	2.212	5.431	14.004
Protein yield (kg)	322.2	57.63	137.86	467.03	17.89
Protein percentage (%)	2.97	0.192	2.199	3.538	6.465

Table S2-1. Primers and procedures for PCR used in SNP identification.

Primer ID	Location	Primer sequence (5'-3')	Product length (bp)	Annealing temp. (°C)
LPIN1-1F<br>*LPIN1-1R*	5′ flanking region	ggagttatttctccactgatctcc<br>agcaatgcctaccagcaaat	643	60
LPIN1-2F<br>*LPIN1-2R*	5′ flanking region	aatgagaaggcccatggttt<br>agtccacagggtcccaaag	800	60
LPIN1-3F<br>*LPIN1-3R*	5′ flanking region	tcctgtaattactcatcatttttgtct<br>cgctcagtaatccaactgattg	638	60
LPIN1-4F<br>*LPIN1-4R*	5′ flanking region	tggataaagtcattcctccctta<br>cgggtcgatcttcctctttc	521	60
LPIN1-5F<br>*LPIN1-5R*	Exon1	agagggtgcaggtgaaagg<br>caaacacgcaccatggttta	609	60
LPIN1-6F<br>*LPIN1-6R*	Exon2	taaacccaggactcctcatctg<br>aagctggctttgtgggaag	580	60
LPIN1-7F<br>*LPIN1-7R*	Exon3	gtgaccccatggacagtagc<br>tgctttacgccactataaatcttg	582	60
LPIN1-8F<br>*LPIN1-8R*	Exon4	gcattggaagcatggagtct<br>acctgcacttctcctgcatc	583	60
LPIN1-9F<br>*LPIN1-9R*	Exon5	aatggcatttagtgacatgttga<br>agcacagggaaccctactca	703	60
LPIN1-10F<br>*LPIN1-10R*	Exon6	tctgagtaatgtttttcgtttcca<br>tcactgttttctccccctga	585	60
LPIN1-11F<br>*LPIN1-11R*	Exon7	cgtgttctgtccgctcct<br>agcagcagcagcttgaaata	463	60
LPIN1-12F<br>*LPIN1-12R*	Exon8	catttgctttggaaacacga<br>tcccctctgaagaatcctga	611	60
LPIN1-13F<br>*LPIN1-13R*	Exon9	ggaagtgtaggggttaaaaatgg<br>ctcaaacacgcctgacgac	659	60
LPIN1-14F<br>*LPIN1-14R*	Exon10	tgattaagggtgcacttgtcc<br>atagaggccagggctgtga	587	60
LPIN1-15F<br>*LPIN1-15R*	Exon11	ggagtttggctggaggaag<br>accccatagactgtggccta	505	60
LPIN1-16F<br>*LPIN1-16R*	Exon12	tggatgccattaggttttgc<br>ctgaccacactctccaagca	503	60
LPIN1-17F	Exon13	agctcgattgtagacgacagc	501	60

LPIN1-17R		tttggaaagtcagtatctatggagtg		
LPIN1-18F	Exon14	agagtgatgatgccagcaag	517	60
LPIN1-18R		acaggaggcaccgaaatg		
LPIN1-19F	Exon15	ccaagtcctggaaagaacca	500	60
LPIN1-19R		atgtcagggagccactgc		
LPIN1-20F	Exon16	ttgaagtgtagttgatttacagtgttg	501	60
LPIN1-20R		accatcttccctccccatc		
LPIN1-21F	Exon17	ccatgaggattaaaccaagtga	560	60
LPIN1-21R		tttcccaagggccatgat		
LPIN1-22F	Exon18	cagacttgaaagtgcggattc	520	60
LPIN1-22R		tttcaagctgaaacccacaa		
LPIN1-23F	Exon19	ttatgcgattggctttactgg	471	60
LPIN1-23R		ccttctatgtgccctgaagtg		
LPIN1-24F	Exon20	tttcctataagttagcgagggact	880	60
LPIN1-24R		ttagctttaactggaagaatgagaga		
LPIN1-25F	3′ flanking region	cttgggatgacgagatccag	822	60
LPIN1-25R		gcccttaatctgagtgggaaa		
LPIN1-26F	3′ flanking region	actctggggttactgttaataagcat	720	60
LPIN1-26R		taaaattcaccccaaaataccc		
LPIN1-27F	3′ flanking region	ccctcctgatggtccatgt	700	60
LPIN1-27R		cacgtgtgatgaacatttgatg		
LPIN1-28F	3′ flanking region	cccggagaggctgtaacttt	545	60
LPIN1-28R		agtatgcttgataccatacaacagga		

Table S2-2. Reaction system.

Content	Dosage	Total
Forward primer (10 pmol/μL)	1.25 μL	
Reverse primer (10 pmol/μL)	1.25 μL	
2 × Taq Master Mix	12.5 μL	25 μL
DNA (50-100 ng/μL)	2 μL	
ddH_2O	8 μL	

Table S2-3. The procedures of PCR amplification.

Temperature (°C)	Time	Cycle No.
95	5 min	1
95	30 s	
Annealing Temp.	30 s	35
72	40 s	
72	10 min	1
4	Forever	1

Table S3. Additive, dominant and allele substitution effects of seven SNPs on milk production traits of *LPIN1* gene in Chinese Holstein.

SNP	Lactation	Genetic effects	Milk yield (kg)	Fat yield (kg)	Fat percentage (%)	Protein yield (kg)	Protein percentage (%)
g.86129263C>G	1	Additive effect (a)	−74.85	**−4.2***	−0.0132	**−2.94***	−0.0064
		Dominant effect (d)	20.73	−0.01	−0.0156	0.63	−0.0026
		Substitution effect (α)	−65.58	−4.21	−0.0201	−2.66	−0.0075
	2	Additive effect (a)	−98.44	−1.06	0.0333	−2.58	0.0017
		Dominant effect (d)	−20.01	−5.26	−0.0417	−1.66	−0.0116
		Substitution effect (α)	−107.39	−3.41	0.0146	−3.32	−0.0035
c.637T>C	1	Additive effect (a)	22.78	0.14	−0.0052	1.42	0.0076
		Dominant effect (d)	15.55	1.2	0.0078	0.53	0.0016
		Substitution effect (α)	20.25	−0.05	−0.0065	1.33	0.0073
	2	Additive effect (a)	63.35	−1.69	**−0.0442***	0.99	−0.0074
		Dominant effect (d)	**155.91***	3.78	−0.0224	**5.22****	0.0051
		Substitution effect (α)	37.98	−2.31	**−0.0406***	0.14	−0.0082
c.708A>G	1	Additive effect (a)	8.98	0.62	0.0025	−0.33	−0.0066
		Dominant effect (d)	−14.04	1.23	0.0149	−0.04	0.0036

		Substitution effect (α)	11.43	0.41	−0.0001	−0.32	−0.0073
		Additive effect (a)	−57.63	1.57	0.0431	−0.33	0.0111
	2	Dominant effect (d)	**177.45****	**4.89***	−0.0191	**5.8****	0.0031
		Substitution effect (α)	−88.56	0.71	0.0464	−1.34	0.0106
c.1521C>T		Additive effect (a)	−87.78	**−4.19***	−0.0082	**−2.93***	−0.0021
	1	Dominant effect (d)	−25.1	−0.17	−0.0028	0.09	0.0059
		Substitution effect (α)	−98.97	−4.27	−0.0095	−2.89	0.0006
		Additive effect (a)	**−263.84****	0.79	**0.0503***	−1.88	0.0104
	2	Dominant effect (d)	**−305.32****	−5.1	−0.0199	−3.12	0.0075
		Substitution effect (α)	**−400.01****	−1.48	0.0414	−3.28	0.0138
c.1555A>C		Additive effect (a)	14.57	0.77	0.0015	−0.29	−0.0078
	1	Dominant effect (d)	−27.76	-0.49	0.0058	−0.74	0.0016
		Substitution effect (α)	19.46	0.85	0.0004	−0.16	−0.0081
		Additive effect (a)	−81.8	1.51	**0.0499****	−0.88	**0.013***
	2	Dominant effect (d)	**184.66****	**5.05***	−0.0183	**5.51****	0.0009
		Substitution effect (α)	**−114.34***	0.62	**0.0531****	−1.85	0.0128
g.86049523C>T		Additive effect (a)	−108.19	3.58	0.0559	-0.65	0.0209
	1	Dominant effect (d)	211.57	**17.73****	0.0823	**8.99***	0.0181
		Substitution effect (α)	57.6	**17.48***	0.1203	6.4	0.035
		Additive effect (a)	**434.67***	**18.53****	0.0394	**13.58****	−0.0046
	2	Dominant effect (d)	278.98	**15.55***	0.0747	**12.02***	0.0293
		Substitution effect (α)	**653.28***	**30.71***	0.0979	**23***	0.0183
g.86049389C>T		Additive effect (a)	−98.9	0.87	0.0332	−0.27	**0.0218****
	1	Dominant effect (d)	**143.22***	**7.6****	0.0178	**7.18****	**0.0208***
		Substitution effect (α)	−26.83	4.7	0.0422	3.35	**0.0323****
		Additive effect (a)	−66.75	−3.84	−0.0175	−3.66	−0.0172
	2	Dominant effect (d)	−124.29	**−10.63****	−0.0525	−4.47	−0.0056
		Substitution effect (α)	−129.28	**−9.18***	−0.044	−5.91	−0.02

Note: * indicates $p < 0.05$; ** indicates $p < 0.01$.
